# Supplementary figures and images for: High prevalence of small intestine bacteria overgrowth and asymptomatic carriage of enteric pathogens in stunted children in Antananarivo, Madagascar
Source: PLoS Negl Trop Dis. 2022 May 9;16(5):e0009849. doi: 10.1371/journal.pntd.0009849 (PMC9119516; doi:10.1371/journal.pntd.0009849)

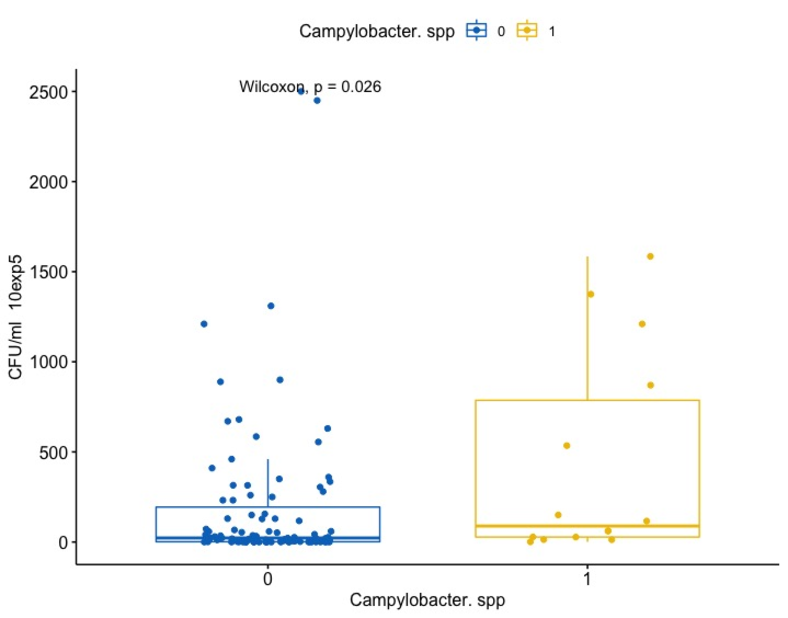

Supplement: S1 Fig — Distributions of the CFU in duodenal aspirates with SIBO matching with feces not contaminated by Campylobacter spp. [0 (in blue)]; and in duodenal aspirates with SIBO matching with feces contaminated by Campylobacter spp. [1 (in yellow)]. (TIF) [file pntd.0009849.s001.tif]

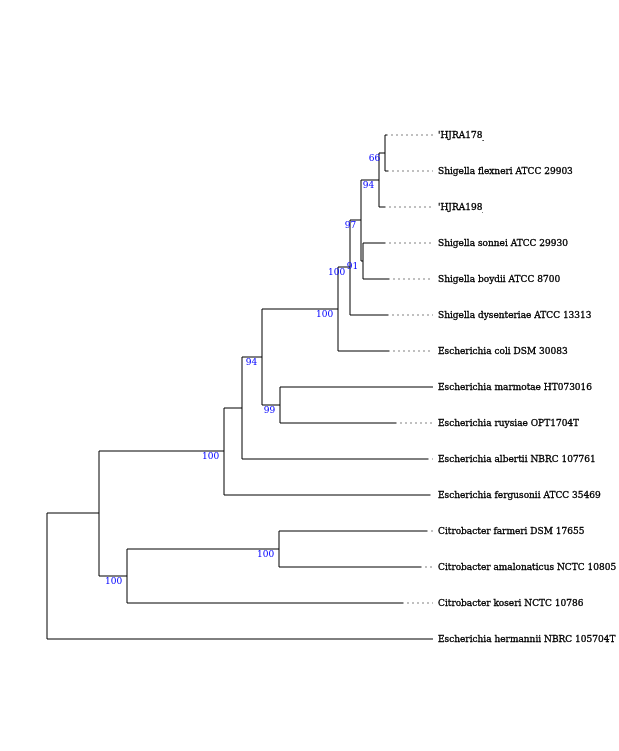

Supplement: S2 Fig — The phylogenomic tree includes the two Shigella isolates from HJRA178—HJRA198 samples constructed on the Type Strain Genome Server (TYGS) (at https://tygs.dsmz.de) using FastME from the genome blast distance phylogeny (GBDP). [26,27]. (TIF) [file pntd.0009849.s002.tif]

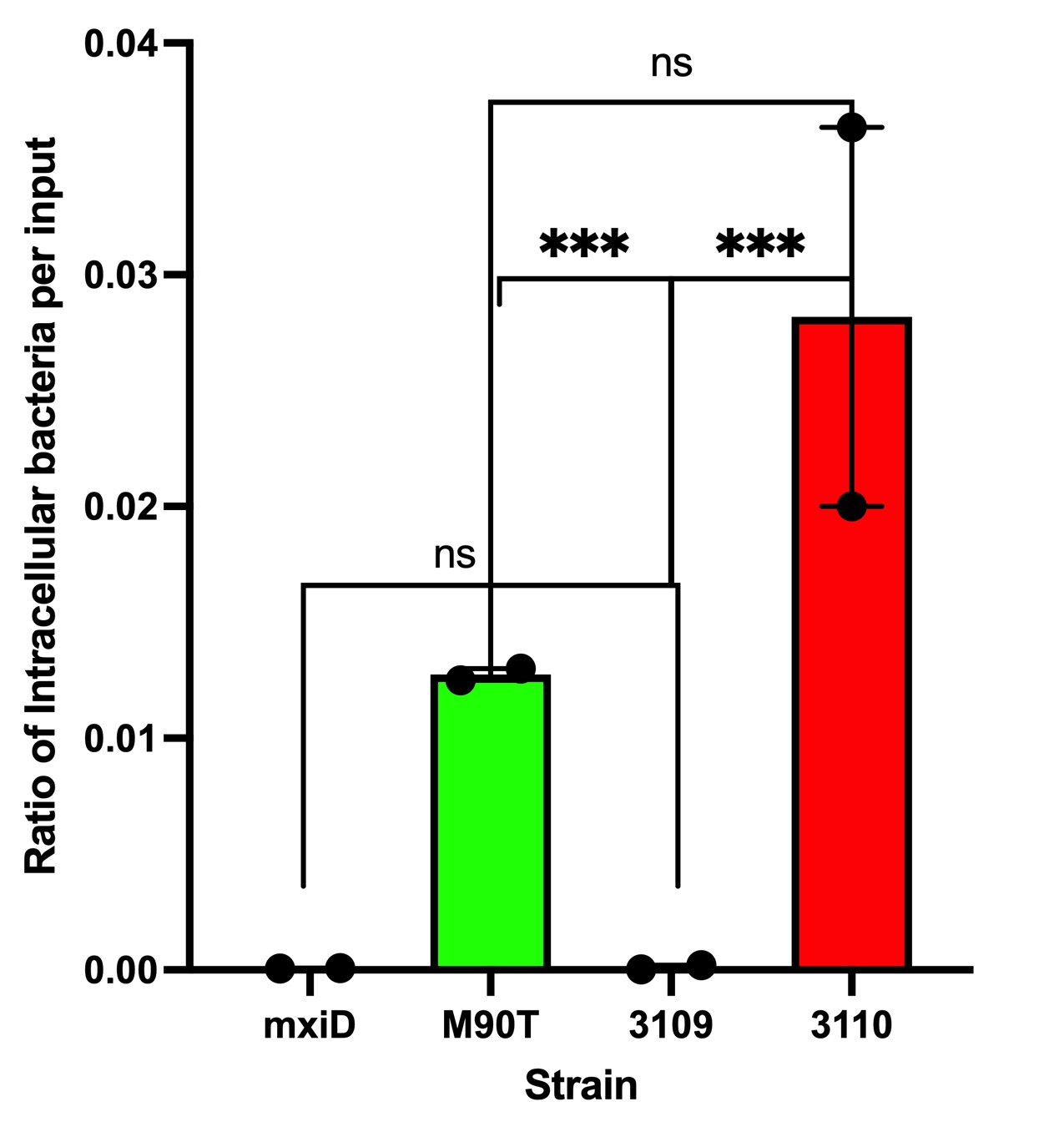

Supplement: S3 Fig — (TIF) [file pntd.0009849.s003.tif]
