# Supplementary material for: High prevalence of small intestine bacteria overgrowth and asymptomatic carriage of enteric pathogens in stunted children in Antananarivo, Madagascar
Source: PLoS Negl Trop Dis. 2022 May 9;16(5):e0009849. doi: 10.1371/journal.pntd.0009849 (PMC9119516; doi:10.1371/journal.pntd.0009849)
Supplement: S3 Table — (PDF) [file pntd.0009849.s006.pdf]

S3 Table. Bivariate analysis for stomach pH and SIBO.

```
 ttest ph_estomac, by(SIBO)

Two-sample t test with equal variances
------------------------------------------------------------------------------
   Group |     Obs        Mean    Std. Err.   Std. Dev.   [95% Conf. Interval]
---------+--------------------------------------------------------------------
      No |      21    2.142857    .3604608    1.651839    1.390949    2.894765
     Yes |      79    2.646835    .2462167    2.188421    2.156656    3.137015
---------+--------------------------------------------------------------------
combined |     100       2.541    .2089758    2.089758    2.126347    2.955653
---------+--------------------------------------------------------------------
    diff |           -.5039783    .5131575               -1.522323     .514366
------------------------------------------------------------------------------
    diff = mean(No) - mean(Yes)                                   t =  -0.9821
Ho: diff = 0                                     degrees of freedom =       98

    Ha: diff < 0                 Ha: diff != 0                 Ha: diff > 0
 Pr(T < t) = 0.1642         Pr(|T| > |t|) = 0.3285          Pr(T > t) = 0.8358
```
