# Supplementary material for: High prevalence of small intestine bacteria overgrowth and asymptomatic carriage of enteric pathogens in stunted children in Antananarivo, Madagascar
Source: PLoS Negl Trop Dis. 2022 May 9;16(5):e0009849. doi: 10.1371/journal.pntd.0009849 (PMC9119516; doi:10.1371/journal.pntd.0009849)
Supplement: S1 Text — (PDF) [file pntd.0009849.s011.pdf]

AFRIBIOTA Investigators (Group authorship in alphabetical order): Emilson Jean Andriatahirintsoa, Centre Hospitalier Universitaire Mère Enfant de Tsaralalana, Antananarivo, Madagascar ; Laurence Barbot-Trystram, Hôpital Pitié-Salpêtrière, Paris, France; Robert Barouki, Hôpital Necker- Enfants maladies, Paris, France ; Alexandra Bastaraud, Institut Pasteur de Madagascar, Antananarivo, Madagascar ; Jean-Marc Collard, Institut Pasteur de Madagascar, Antananarivo, Madagascar ; Maria Doria, Institut Pasteur, Paris, France ; Darragh Duffy, Institut Pasteur, Paris, France ; Aurélie Etienne, Institut Pasteur, Paris, France/ Institut Pasteur de Madagascar, Madagascar ; Serge Ghislain Djorie, Institut Pasteur de Bangui, Bangui, Central African Republic ; Tamara Giles-Vernick, Institut Pasteur, Paris, France ; Bolmbaye Privat Gondje, Complexe Pédiatrique de Bangui, Bangui, Central African Republic ; Jean-Chrysostome Gody, Complexe Pédiatrique de Bangui, Bangui, Central African Republic ; Milena Hasan, Institut Pasteur, Paris, France ; Jean-Michel Héraud, Institut Pasteur de Madagascar, Antananarivo, Madagascar ; Francis Allan Hunald, Centre Hospitalier Universitaire Joseph Ravoahangy Andrianavalona (CHU-JRA), Antananarivo, Madagascar ; Nathalie Kapel, Hôpital Pitié-Salpêtrière, Paris, France ; Jean-Pierre Lombart, Institut Pasteur de Bangui, Bangui, Central African Republic ; Alexandre Manirakiza, Institut Pasteur de Bangui, Bangui, Central African Republic ; Synthia Nazita Nigatoloum, Complexe Pédiatrique de Bangui, Bangui, Central African Republic ; Laura Wegener Parfrey, University of British Columbia, Vancouver, Canada ; Lisette Raharimalala, Centre de Santé Materno-Infantile, Tsaralalana, Antananarivo, Madagascar ; Maheninasy Rakotondrainipiana, Institut Pasteur de Madagascar, Antananarivo, Madagascar ; Rindra Vatosoa Randremanana, Institut Pasteur de Madagascar, Antananarivo, Madagascar ; Harifetra Mamy Richard Randriamizao, Centre Hospitalier Universitaire Joseph Ravoahangy Andrianavalona (CHU-JRA), Antananarivo, Madagascar ; Frédérique Randrianirina, Institut Pasteur de Madagascar, Antananarivo, Madagascar ; Annick Lalaina Robinson, Centre Hospitalier Universitaire Mère Enfant de Tsaralalana, Antananarivo, Madagascar ; Pierre-Alain Rubbo, Institut Pasteur de Bangui, Bangui, République Centrafricaine ; Philippe Sansonetti, Institut Pasteur, Paris, France ; Laura Schaeffer, Institut Pasteur, Paris, France ; Ionela Gouandjika-Vassilache, Instiut Pasteur de Bangui, Bangui, République Centrafricaine ; Pascale Vonaesch, Institut Pasteur, Paris, France ; Sonia Sandrine Vondo, Complexe Pédiatrique de Bangui, Bangui, Central African Republic ; Inès Vigan-Womas, Institut Pasteur de Madagascar, Antananarivo, Madagascar.
