# Supplementary material for: High prevalence of small intestine bacteria overgrowth and asymptomatic carriage of enteric pathogens in stunted children in Antananarivo, Madagascar
Source: PLoS Negl Trop Dis. 2022 May 9;16(5):e0009849. doi: 10.1371/journal.pntd.0009849 (PMC9119516; doi:10.1371/journal.pntd.0009849)
Supplement: Attachment — Submitted filename: 2022 03 Responses to the editor.pdf [file pntd.0009849.s013.pdf]

23.02.2022
Decision on your PLOS Neglected Tropical Diseases submission (PNTD-D-21-01414R1) –

Dear Dr Collard,

Thank you very much for submitting your manuscript "High prevalence of small intestine bacteria overgrowth and asymptomatic carriage of enteric pathogens in stunted children in Antananarivo, Madagascar." for consideration at PLOS Neglected Tropical Diseases. As with all papers reviewed by the journal, your manuscript was reviewed by members of the editorial board and by several independent reviewers. The reviewers appreciated the attention to an important topic. Based on the reviews, we are likely to accept this manuscript for publication, providing that you modify the manuscript according to the review recommendations.

Please prepare and submit your revised manuscript within 30 days. If you anticipate any delay, please let us know the expected resubmission date by replying to this email.

When you are ready to resubmit, please upload the following:

[1] A letter containing a detailed list of your responses to all review comments, and a description of the changes you have made in the manuscript.
Please note while forming your response, if your article is accepted, you may have the opportunity to make the peer review history publicly available. The record will include editor decision letters (with reviews) and your responses to reviewer comments. If eligible, we will contact you to opt in or out

[2] Two versions of the revised manuscript: one with either highlights or tracked changes denoting where the text has been changed; the other a clean version (uploaded as the manuscript file).

Important additional instructions are given below your reviewer comments.

Thank you again for your submission to our journal. We hope that our editorial process has been constructive so far, and we welcome your feedback at any time. Please don't hesitate to contact us if you have any questions or comments.

Sincerely,

Andrew S. Azman
Deputy Editor
PLOS Neglected Tropical Diseases

****

Reviewer's Responses to Questions

Key Review Criteria Required for Acceptance?</br></br>
As you describe the new analyses required for acceptance, please consider the following:</br></br>

Methods</br></br>
-Are the objectives of the study clearly articulated with a clear testable hypothesis stated?</br>
-Is the study design appropriate to address the stated objectives?</br>
-Is the population clearly described and appropriate for the hypothesis being tested?</br>
-Is the sample size sufficient to ensure adequate power to address the hypothesis being tested?</br>
-Were correct statistical analysis used to support conclusions?</br>
-Are there concerns about ethical or regulatory requirements being met?</br></br>

Reviewer #1: yes

Reviewer #2: -If the samples from ref 15 (153 fecal and 12 duodenal) were included in the data set used here then this needs to be explicitly stated so readers understand the duplication of published data. If there is overlap, I would add a sentence stating that "153 fecal sample and 12 duodenal samples utilized included in this analysis have been previously analyzed and published on"(with appropriate reference).

We present here the results of an extension of the initial dataset, including the original data on SIBO presented in PNAS but extending the findings to a much larger set of duodenal samples (109 selected on 165 samples compared to 12 duodenal samples in the initial PNAS paper Vonaesch et al., 2018). We also stressed that previously, none of the 153 fecal samples (those mentioned in the PNAS paper) were screened by qPCR for the different targeted pathogens (specific markers), even if some of them were analyzed by a metataxonomic approach which is obviously less sensitive (V4 region of the rDNA) than our qPCR approach. In our paper, we screened 464 fecal samples - by qPCR - (264 controls -C-, 104 MS and 96 SS children) versus 153 - by metataxonomic analysis - (69 controls -C-, 46 MS and 38 SS children) in the PNAS paper.

This has been again rephrased in the MS to make it clearer as recommended by reviewer #2.
Lines 338-341:
In our study, a total of 165 duodenal aspirates were collected; … This is a substantial extension of the initial dataset presented in ref. 15 (12 duodenal samples).

-It is mentioned in the methods that time between defecation and freeing was tracked. I would report the average either in the methods or in the first paragraph of the results.

This has been mentioned in the MS as recommended by reviewer #2.
Lines 167-168: The time for freezing the emitted feces was comprised between 6 min and 23.66h with a mean of 3.69h.

-When authors state the first ml of aspirate was discarded for "flashing" out of possible contamination, do you mean 'flushing'?
Line 178: Sorry, it was a typo. Of course, we meant flushing. Corrected in the MS

Results</br></br>
-Does the analysis presented match the analysis plan?</br>

-Are the results clearly and completely presented?</br>
-Are the figures (Tables, Images) of sufficient quality for clarity?</br></br>

Reviewer #1: Yes

Reviewer #2: -All presented data and supplementary information (i.e. Supplementary tables S1 and S2) need to be in English as this is an English journal. Please translate.

Translated and supplied as S1 and S2

-The finding of SIBO's association with Campylobacter potentially quite significant. Neither in the manuscript nor in table S4 is directionality shown. Would add average CFU/ml in Campy positive and negative subjects with SD to the body of the manuscript (line 347) so readers can understand this relationship.

As requested, we added a Fig (S1 Fig.) representing the distributions of the CFU in duodenal aspirates with SIBO matching with feces contaminated or not by *Campylobacter* spp.

Lines 385-388: The only pathogen significantly associated with the duodenal CFU count was *Campylobacter* spp. (p=0.026) (S5 Table). The distributions of the CFU in duodenal aspirates with SIBO matching with feces contaminated or not by *Campylobacter* spp. are presented in S1 Fig.

Conclusions</br></br>
-Are the conclusions supported by the data presented?</br>
-Are the limitations of analysis clearly described?</br>
-Do the authors discuss how these data can be helpful to advance our understanding of the topic under study?</br>
-Is public health relevance addressed?</br></br>

Reviewer #1: Yes

Reviewer #2: -Line 435 – 437: This is the first mention of pH values being the same between stunted and control children. This, and the reference to S7 Table, should be moved to the results section as new results should not be first presented in the discussion.

The sentence in the discussion section was slightly modified (bold characters) Lines 453-454:
However, the pH values measured **in the stomach samples from** stunted children were low and there were no significant differences in a bivariate analysis for pH and SIBO (S3 Table).
and this was, as recommended by reviewer #2, introduced in the results section, in the paragraph on SIBO and risk factors.
Lines 359-361: Regarding the pH values measured in the stomach samples from stunted children, they were low and there were no significant differences in a bivariate analysis for stomach pH and SIBO (S3 Table).

We also recall to the reviewer that SIBO was determined in stunted children only [Remark made by reviewer #2 *Line 435 – 437: This is the first mention of pH values being the same between stunted and control children*].
Consecutively, the order of Supplementary Tables changed:
S7 -> S4
S4 -> S5
S5 -> S6
S6 -> S7

Editorial and Data Presentation Modifications?</br>
Use this section for editorial suggestions as well as relatively minor modifications of existing data that would enhance clarity. If the only modifications needed are minor and/or editorial, you may wish to recommend “Minor Revision” or “Accept”.

Reviewer #1: the authors carefully responded to the reviewer's comments.

Reviewer #2: -Issues with grammar and typos remain but much improved.

We again re-read the MS for grammar and typos

-Abbreviations should be defined at their first use (see line 114: SARI/ILI).

We added the definition of the acronym at lines 140-141: **severe acute respiratory infections** (SARI)/ influenza-like illness (ILI)

Summary and General Comments</br></br>
Use this section to provide overall comments, discuss strengths/weaknesses of the study, novelty, significance, general execution and scholarship. You may also include additional comments for the author, including concerns about dual publication, research ethics, or publication ethics. If requesting major revision, please articulate the new experiments that are needed.

Reviewer #1: This study includes a substantial amount of data regarding intestinal overgrowth and makes some interesting observations about relationship with oral organisms. The stool microbiology is interesting, but is largely confirmatory. It is interesting that the apparent "pathogens" did not significantly relate to the degree of malnutrition.

Reviewer #2: Authors have adequately addressed concerns in the first manuscript with a few minor suggestions remaining. I continue to think that this is a unique and important analysis with potentially large impact on the field of environmental enteric dysfunction and malnutrition.

We again thank the 2 reviewers for their major contributions in improving our manuscript and for their overall positive evaluation

----

PLOS authors have the option to publish the peer review history of their article (what does this mean?). If published, this will include your full peer review and any attached files.

If you choose “no”, your identity will remain anonymous but your review may still be made public.

Do you want your identity to be public for this peer review? For information about this choice, including consent withdrawal, please see our Privacy Policy.

Reviewer #1: No

Reviewer #2: No

Figure Files:
While revising your submission, please upload your figure files to the Preflight Analysis and Conversion Engine (PACE) digital diagnostic tool, https://pacev2.apexcovantage.com. PACE helps ensure that figures meet PLOS requirements. To use PACE, you must first register as a user. Then, login and navigate to the UPLOAD tab, where you will find detailed instructions on how to use the tool. If you encounter any issues or have any questions when using PACE, please email us at figures@plos.org.

Data Requirements:
Please note that, as a condition of publication, PLOS' data policy requires that you make available all data used to draw the conclusions outlined in your manuscript. Data must be deposited in an appropriate repository, included within the body of the manuscript, or uploaded as supporting information. This includes all numerical values that were used to generate graphs, histograms etc.. For an example see here: http://www.plosbiology.org/article/info%3Adoi%2F10.1371%2Fjournal.pbio.1001908#s5.

Reproducibility:
To enhance the reproducibility of your results, we recommend that you deposit your laboratory protocols in protocols.io, where a protocol can be assigned its own identifier (DOI) such that it can be cited independently in the future. Additionally, PLOS ONE offers an option to publish peer-reviewed clinical study protocols. Read more information on sharing protocols at https://plos.org/protocols?utm_medium=editorial-email&utm_source=authorletters&utm_campaign=protocols

References
Please review your reference list to ensure that it is complete and correct. If you have cited papers that have been retracted, please include the rationale for doing so in the manuscript text, or remove these references and replace them with relevant current references. Any changes to the reference list should be mentioned in the rebuttal letter that accompanies your

revised manuscript. If you need to cite a retracted article, indicate the article's retracted status in the References list and also include a citation and full reference for the retraction notice.

In compliance with data protection regulations, you may request that we remove your personal registration details at any time. (Use the following URL: https://www.editorialmanager.com/pntd/login.asp?a=r). Please contact the publication office if you have any questions.
