# Supplementary material for: High prevalence of small intestine bacteria overgrowth and asymptomatic carriage of enteric pathogens in stunted children in Antananarivo, Madagascar
Source: PLoS Negl Trop Dis. 2022 May 9;16(5):e0009849. doi: 10.1371/journal.pntd.0009849 (PMC9119516; doi:10.1371/journal.pntd.0009849)
Supplement: Attachment — Submitted filename: Responses to the reviewers.docx [file pntd.0009849.s012.docx]

**Responses to Reviewer's Questions**

        **Key Review Criteria Required for Acceptance**
        As you describe the new analyses required for acceptance, please consider the following

        Methods
        -Are the objectives of the study clearly articulated with a clear testable hypothesis stated >
        -Is the study design appropriate to address the stated objectives?
        -Is the population clearly described and appropriate for the hypothesis being tested?
        -Is the sample size sufficient to ensure adequate power to address the hypothesis being tested?
        -Were correct statistical analysis used to support conclusions?
        -Are there concerns about ethical or regulatory requirements being met?

        Reviewer #1: The methods are well described and are appropriate.

We thank the reviewer for a positive evaluation of this manuscript section.

        Reviewer #2: -Enrollment criteria need to be reviewed in detail.  “other severe diseases” is vague.

The manuscript was adapted to better describe the enrollment criteria. We now specified that other severe diseases encompass the following:

- dysenteric syndrome, choleriform syndrome, gastroenteritis,
- acute respiratory infections: bronchiolitis, pneumonia
- any type of viral, bacterial or parasitic serious infections: meningitis, malaria, acute otitis media,varicella, measles
The sentence in the revised manuscript now reads as follows (Lines 206-209): “Briefly the study population comprises HIV-negative children aged 2 to 5 years, neither suffering from acute malnutrition, nor from any other severe diseases (such as dysenteric syndrome, SARI/ILI, meningitis, malaria, acute otitis media, varicella, measles, …) who were recruited in the community of the two districts …”

-Were all children truly admitted to the hospital as stated in line 119?  If so, reason for admission needs to be detailed.

Yes, all children were sent to the hospital for sample collection. The text was adapted accordingly and clarified. See line 119, which correspond to lines 212-216 in the revised manuscript and now read as follows: “Children were recruited either in the community (community-recruited children) or directly in the hospital (hospital-recruited children). The caregivers of all children received oral and written explanations of the study and provided written consent to participate. Anthropometric measurements were collected at the community-health centers and in the hospital. All children were admitted to the hospital for sample collection.

-It seems that duodenal sampling technique via nasogastric tube could introduce nasopharyngeal flora into the sample thus contaminating it and skewing results for an over-representation of oral taxa.  Authors should address this in methods and/or the limitations section of the discussion.  
We think that due to our sampling technique it is unlikely that contamination has occurred, but agree with the reviewer that this should be directly addressed in the manuscript. The first ml of aspiration from both stomach and duodenum were discarded in order to flash out possible contaminating bacteria and carry-over from the more proximal compartments. Therefore, it seems unlikely that duodenal sampling technique via nasogastric tube could introduce nasopharyngeal flora into the sample thus contaminating it. We added this clarification in the ‘Limitation section’ of the manuscript (line 756-758): “Regarding the duodenal sampling technique via nasogastric tubes, we ensured that the first ml of aspiration from both the stomach and the duodenum were discarded. This allowed flashing out possible contaminating bacteria and minimize the carry-over from the more proximal compartments.” This was also added in the M&M section (Lines 251-253)

-Data collection on co-variates is unclear.  A complete list of what was assessed in univariate analysis needs to be provided with details (ie. Who assessed for dental carries).  If extensive then perhaps a correction for multiple comparisons is appropriate. 
We now provide two files as supplementary data: one which lists all data (risk factors) collected and used in the univariate analysis for risk factors associated with SIBO, and another summarizing the results for the univariate and multivariate analyses (Lines 315-316).

S1 Table. Lists of all data (risk factors) collected and used in the univariate analysis with SIBO.

(XLSX)

S2 Table. Results for the uni- and multivariate analysis.

(XLSX)

As the data were collected in a French speaking country, the data presented in the two files are in French.

We are now referring to these two files in the Material and methods chapter:

“For risk factors analysis, bivariate logistic regression analysis was carried out between different socio-economic and clinical factors (see Tables S1 and S2) and the presence of SIBO using the Chi-squared test or the Fisher’s exact test. Variables displaying a p-value of ≤0.25 were included in the multivariate logistic regression model.”

-Can authors cite a reference for using a CT cutoff of 37?  This seems higher than most qPCR analyses.  How was this value chosen?
A Ct cutoff of 37 or 38 is routinely used in real-time PCR assays, see the following references: <https://pubmed.ncbi.nlm.nih.gov/33650802/>, <https://pubmed.ncbi.nlm.nih.gov/30031012/>, <https://pubmed.ncbi.nlm.nih.gov/31097205/>.

We also established standard curves for each target ensuring primers are efficient and will result in a proportional dose-response curve with a R^2^ of the curve which should be > 0.99 to provide a good confidence within the correlation. Besides knowing that our primers are efficient, the standard curve tells us about the detection limit and the linearity ensures about the CT cutoff value of 37 is applicable.

It is explained in the MS (lines 350-351)

Hereafter an example:

-eae, as authors state, could represent EHEC as well as EPEC. When authors use “either target” this may over-represent EPEC with some of these isolates being EHEC.
We fully agree with the reviewer’s comment.

We have therefore modified the corresponding sentences in the material and methods and results’ sections, which now read as follows:

Lines 363-366: “When one of the two targets was detected for ETEC (*estIa* or *eltB*) or EAEC (*aggR* or *aaiC*), the pathogen was considered to be present. Since *eae* can be present both in EPEC and EHEC, we considered only the presence of *bfpA* gene for EHEC detection.”

AND

Lines 478 -> 480. We considered a sample to be infected by ETEC or EAEC if at least one of the two genes was present, and by EAEC when *bfpA* gene was detected. Based on this definition, ETEC and EAEC were present in about one-third (ETEC=32.8 and EAEC=29.9%) of the children (C, MS and SS children) and EPEC in 14% of the children.”

Supporting results (data not shown under this format but can be found in S3 Table)

|  | **% for C = controls (N=264)** | **% for MS = moderately stunted (N=104)** | **% for SS = severely stunted (N=96)** | **% for stunted children = MS+SS (N=200)** | **Total in % for C + MS + SS (N=464)** |
| --- | --- | --- | --- | --- | --- |
| *Salmonella* spp. | 7.95 (N=21) | 5.21 (N=5) | 12.5 (N=12) | 8.5 (N=17) | 8.19 (N=38) |
| *Shigella* spp. | 80.68 (N=213) | 90.38 (N=94) | 84.38 (N=81) | 87.5 (N=175) | 83.62 (N=388) |
| ETEC *estIa* | 4.17 (N=11) | 5.77 (N=6) | 6.25 (N=6) | 6.0 (N=12) | 4.96 (N=23) |
| ETEC *eltB* | 29.92 (N=79) | 40.38 (N=42) | 32.29 (N=31) | 36.5 (N=73) | 32.76 (N=152) |
| ETEC *estIa*  + *eltB* | 3.03 (N=8) | 0.96 (N=2) | 4.17 (N=4) | 3.00 (N=6) | 3.02 (N=14) |
| EPEC *bfpA* | 13.26 (N=35) | 11.54 (N=12) | 18.75 (N=18) | 15.0 (N=30) | 14.01 (N=65) |
| EPEC *eae* | 37.50 (N=99) | 36.54 (N=38) | 36.46 (N=35) | 36.5 (N=73) | 37.07 (N=172) |
| EPEC *bfpA* + *eae* | 12.88 (N=34) | 11.54 (N=12) | 17.71 (N=17) | 14.50 (N=29) | 13.58 (N=63) |
| EAEC *aggR* | 31.06 (N=82) | 27.88 (N=29) | 29.17 (N=28) | 28.5 (N=57) | 29.96 (N=139) |
| EAEC *aaiC* | 17.80 (N=47) | 25.96 (N=27) | 22.92 (N=22) | 24.5 (N=49) | 20.69 (N=96) |
| EAEC *aggR* + *aaiC* | 11.74 (N=31) | 5.77 (N=16) | 11.46 (N=11) | 13.50 (N=27) | 12.50 (N=58) |
| *Campylobacter jejuni/coli* | 14,39 (N=38) | 11,54 (N=12) | 13.54 (N=13) | 12.5 (N=25) | 13.58 (N=63) |
| *Vibrio cholerae* | 0 (N=0) | 0 (N=0) | 0 (N=0) | 0 (N=0) | 0 (N=0) |

        --------------------

<b>Results</b></br></br>
-Does the analysis presented match the analysis plan?</br>
-Are the results clearly and completely presented?</br>
-Are the figures (Tables, Images) of sufficient quality for clarity?</br></br>

Reviewer #1: Yes
We thank the reviewer for his positive evaluation.

Reviewer #2: -Would add the number of duodenal aspirates to line 287.
We adapted as suggested. Line 287 (now 442-443) reads now as follows: “Duodenal aspirates (N=109) were analyzed using bacteriological techniques and stools were analyzed by qPCR (Fig. 1).”

-Line 289 – 292 “The duodenum and proximal jejunum…” this is more fitting in the background.  Not in the results. 
This sentence was moved to the discussion section – which seems in our eyes even more appropriate than to place it into the introduction. The phrase can now be found on lines 594-597.

-A better description of how colonies and morphotypes were selected for MALDI-TOF is needed.  Were a random number chosen from each sample?  Were all isolates cultures sent for MADLI-TOF.  This has major implications on the results so this needs to be clear.  The description of this should be in the methods, not results.

This has been clarified in the material and methods section, which now reads as follows (lines 284-286): The isolated colonies were counted by morphotype and at the beginning 2-3 colonies for every morphotype were re-isolated. Re-isolated colonies were all identified by MALDI-TOF mass spectrometry (Bruker Biotyper, Bruker Daltonics, Bremen, Germany). As colonies from the same morphotype from the same plate were all identical (same identification), finally only one colony/morphotype was isolated and subcultured.

-Line 306 – 208 suggests no risk factors for SIBO were identified however in Table 3 handwashing is significant.  Please clarify.  Is table 3 univariate only (if so, suggest changing to multivariate final model).  
We thank the reviewer for pointing out this important mistake. The actual value is 0.07 instead of 0.007 and the result in consequence not significant (>0.05). This has now been corrected in table 3.

-Line 316 states no significant difference was found between pathogen carriage amongst stunting groups.  However, in Table 4 Salmonella and Shigella are statistically significant.  Please clarify. 
We considered only values with *p <* 0.05 to be statistically significant. In the Table, the p-values are 0.13 for *Salmonella* and 0.07 for *Shigella*. Both values are > 0.05 and we thus considered them statistically non-significant.

-Line 319 reports mean, median, and range of CT values.  Is this for all pathogens and targets?  
This sentence was clarified and we now mention that it relates to the *ipaH* gene.

The new sentence (lines 480-481) now reads as follows: “The mean, median, lowest and highest *C_T_* values for *ipaH* were 30, 31.5, 14.5 and 45, respectively.”

-Line 320 states no difference in CT values was noted between groups, CT values for which pathogen?  All pathogens?  Please clarify.

The *C_T_* values were not significantly lower in stunted children compared to controls nor for *ipaH* nor for any of the other genes/pathogens (Fig. 3). That was clarified in the MS: Lines 476-477.

-Line 322-323:  Meaning isn’t clear.  1/3 of the children had one of the 3 tested E. coli pathotypes?
This sentence was clarified (Lines 484 -> 486): “ When considering the presence of only one of the two genes for ETEC and EAEC, and only *bfpA* for EPEC since *eae* can be present both in EPEC and EHEC, ETEC and EAEC were present in about one-third (ETEC=32.8 and EAEC=29.9%) of the children (C, MS and SS children) and EPEC in 14% of the children.”

-It is unclear what exactly the cluster dendrogram of Fig 4 was clustered on.  Details need to be provided in the methods section.
We added further details in the methods’ section, which now reads as follows (lines 321-324): “For the clustering analysis, the data consisted in 0s and 1s corresponding to the infection status (detection of the pathogen by qPCR) of the children: 0 = uninfected and 1 = infected. The infection profiles for the children obtained were clustered with a Hierarchical Clustering based on a binary distance coupled with Ward’s agglomeration method. No clustering was applied on the variables.”

-The difference in positivity between conventional and qPCR needs to be explained.  Do you this qPCR has false positives or does traditional PCR miss cases?  I suggest the authors make an argument for a single method and limit reporting to that method.
We agree with the reviewer that the results are puzzling. Based on the literature [i.e. one recent paper: Yang TB, Liu J, Chen J. Compared with conventional PCR assay, qPCR assay greatly improves the detection efficiency of predation. Ecol Evol. 2020 Jun 27;10(14):7713-7722. doi: 10.1002/ece3.6494.] qPCR amplification is more sensitive than conventional PCR (cPCR), which could explain the results obtained. As recommended, we have deleted the part on cPCR [and reference 21] and only kept the qPCR results.

-Analysis on Shigella carriage seems unrelated to the SIBO story.  While interesting, I would suggest removing it and submitting as a separate report.

In order to respect the comment of the reviewer, instead of suppressing the analysis on *Shigella* carriage, which remains for us an important part of this paper, we have substantially reduced the section regarding the *Shigella* carriage. This was also a recommendation of the second reviewer (“The data is important, but it could be said using many fewer lines”.)

-Table 3: Is this univariate or multivariate.  Title should be more descriptive.

The legend of Table 3 has been adapted to more clearly address this point. It now reads as follows: “Possible risk factors associated with SIBO. Only variables having a P-value ≤0.25 in the univariate analysis (S1 Table) were entered into multivariate logistic regression model (S2 Table). All variable were recorded by field workers (clinical research associates). “

-Figure 1:  The abbreviation SR is not defined in the figure legend.  
We thank the reviewer for pointing out this omission. We added SR: Sex Ratio in the legend of Fig. 1.

-Figure 2:  No microbiome analysis was conducted and thus the units on the Y axis of “relative abundance” do not make sense.  Also, the range of 0-500 does not make sense if this is relative abundance.  Please clarify.

We agree with the reviewer that the figure was misleadingly labelled. The Y axis title of Figure 2 was corrected and now reads as “Number of isolates of the 12 most-abundant genera identified by culture techniques in duodenal aspirates of stunted children”. The color code for the different genera is given on the right.

-Figure 4: Legend needs to be expanded to describe what variables were clustered on here.
We have extended the legend and described what variables were clustered on the heatmap as requested by the reviewer.

The legend now reads as follows: “Heatmap showing gene frequency detected by qPCR and height for age Z (HAZ) scores (x-axis: isolates; y-axis: targeted genes). The dendrogram fitted on the isolates (i.e. the subjects) were computed based on an asymmetric binary distance coupled with a Ward agglomeration method based on the presence - black color - or absence of the genes - gray color. The clusters derived from the hierarchical clustering were not shown to be significantly associated to HAZ. No clustering was performed on the variables (targeted genes).”

        --------------------

<b>Conclusions</b></br></br>
-Are the conclusions supported by the data presented?</br>
-Are the limitations of analysis clearly described?</br>
-Do the authors discuss how these data can be helpful to advance our understanding of the topic under study?</br>

-Is public health relevance addressed?</br></br>

Reviewer #1: yes
Many thanks

Reviewer #2: -Authors state they “precisely characterize the bacterial population in the duodenum”.  This is an overstatement as they utilized outdated culture techniques rather than molecular means of quantifying the SIBO dysbiosis.  This should be listed as a limitation and this sentence revised. 
We agree with the Reviewer 2 that this statement is somehow misleading. A paper on the metataxonomic analysis of all duodenal samples (Madagascar and Central African Republic) was recently been submitted to the Journal Gastroenterology [EMID:9fe92c9fb23881d5] (Stunted child growth is associated with small intestinal colonization by oral bacteria driving lipid malabsorption and inflammation by Vonaesch P et al.) but is however still under review. We do not consider culturomics (utilization of a wide range of culturing and incubation conditions) to be an outdated technique as it allows to isolate specific bacterial species for further characterization and confirms the viability of the bacteria present, which is not possible with sequencing alone. According to the comment of the reviewer, we added a sentence in the ‘discussion’ section stressing the limitation of the technique: Lines 559-564 [Corrected sentence]. The sentence now reads as follows: “In this study, duodenal aspirates from stunted children (N=109) were analyzed by cultured-based techniques to investigate SIBO. Although there is currently no single valid test for SIBO [Sachdev AH and Pimentel M. [Gastrointestinal bacterial overgrowth: pathogenesis and clinical significance.](https://pubmed.ncbi.nlm.nih.gov/23997926/) Ther Adv Chronic Dis. 2013. REF 32] and though culture-techniques are limited in the number of taxa that can be identified compared to sequencing techniques, they allow to assess for the viability of the bacteria present and for functional characterization and validation of individual strains. “

-Would discuss differences in positivity rates between this study and others in the literature.  Perhaps culture of duodenal aspirate is more sensitivity than the breath testing utilized in the other studies discussed.

We have added the following sentence (L578 previously L389) in the discussion: “These high SIBO positivity rates in our study also reflect the fact that the cultivation approach is more sensitive than the breath testing utilized in the other studies. “

-Authors suggest that SIBO causes inflammation, villus blunting, and decreased crypt-to-villous ratio.  The relation of SIBO to histology needs to be cited.  Further, stating the relationship is causal is an overstatement.  “Associated” is more appropriate. 
We suppressed part of the text and added the following reference about the relation of SIBO to inflammation:

Adike A, DiBaise JK. Small Intestinal Bacterial Overgrowth: Nutritional Implications, Diagnosis, and Management. Gastroenterology Clinics of North America. 2018, 47:193-208. <https://doi.org/10.1016/j.gtc.2017.09.008> REF36.

Following the reviewer’s recommendation, we have been more cautious in our statements (Line 589), which now read as follows:

“The presence of excessive bacteria in the small intestine is typically ~~described as~~ associated with a malabsorptive syndrome occurring in the context of gut stasis [36]. It was also suggested that SIBO is associated with intestinal inflammation [13] especially in resource-poor communities where an extreme poverty and unhygienic conditions prevail.

We have also modified the text on SIBO in the abstract and the introduction parts (see hereafter).

-Line 399 – 400 “Those genera are generally…”: Needs citation. (now 599)

The following citation was added:

Ref. 37: Caselli E, Fabbri C, D'Accolti M, Soffritti I, Bassi C, Mazzacane S, Franchi M. Defining the oral microbiome by whole-genome sequencing and resistome analysis: the complexity of the healthy picture. BMC Microbiol. 2020 May 18;20(1):120. doi: 10.1186/s12866-020-01801-y.

-Line 406 – 407 “In these stunted children…”:  Sentence is purely speculative and none of this was shown in the cited reference.

We agree with the reviewer that this sentence is speculative but it is one of our hypotheses presented in this ‘discussion’ section and in our previous ref. 14 (now 15). The sentence in the revised manuscript now reads as follows (Lines 605-607): We speculate that in these stunted children, gut maintenance declines and microbes can stray from traditional zones, negatively impacting intestinal homeostasis, host health and altered nutriment absorption [15].

-Line 413:  This is misleading.  Strep, Veillonella, and Rothia are often found in the small intestine as well as the oral cavity.
We agree that it could be misleading and that those genera are found both in the oral cavity and in the small intestine. We deleted in the sentence this part: “normal inhabitants of the human oral cavity. “

The sentence in the revised manuscript now reads as follows (Lines 611-613):

“The three most strongly correlated bacteria with the duodenal inflammatory markers were a *Veillonella* species, a *Streptococcus* species and *Rothia mucilaginosa*.”

-The discussion of URI and children swallowing oral flora is purely speculative and without evidence.  This should be removed.

The text has been removed from the discussion section.

-Again, suggest revising all instances where authors state a causal relationship between pathogen carriage and SIBO/EED.
Revisited as requested by the reviewer.

-Limitations section should be moved to the discussion.  Sampling technique and possible contamination of oral taxa on the nasogastric tube as well as lack of molecular testing on aspirates (i.e. culture techniques) should be added as limitation.

We agreed and moved the ‘Limitations’ section just after the ‘Discussion’ section. We also added several items, notably on the duodenal sampling technique.

--------------------

<b>Editorial and Data Presentation Modifications?</b></br><br/>
Use  this section for editorial suggestions as well as relatively minor modifications of existing data that would enhance clarity. If the only modifications needed are minor and/or editorial, you may wish to recommend “Minor Revision” or “Accept”. 

Reviewer #1: The results section describing the two shigella isolates is too wordy - this can be shortened to very few lines.
Done as recommended by the two reviewers.

Reviewer #2: -There are many typos throughout this manuscript which are irksome to the reviewer
-English and grammar are incorrect throughout the manuscript.  This obscures meaning in places.

We have re-read the manuscript for clarity and English language corrections and the last version was corrected by a native English speaker.

--------------------

<b>Summary and General Comments</b></br></br>
Use  this section to provide overall comments, discuss strengths/weaknesses of the study, novelty, significance, general execution and scholarship. You may also include additional comments for the author, including concerns about dual publication, research ethics, or publication ethics. If requesting major revision, please articulate the new experiments that are needed.

Reviewer #1: This is a well written article that reports high rates of small intestinal overgrowth in stunted, but not acutely ill children aged 2 to 5 years.  The report is consistent with earlier studies in the literature so in this way, the data are mostly confirmatory, but the finding of oral bacteria in the upper small intestine is interesting.  The finding of such a high prevalence of Shigella in fecal samples of all groups is also similar to other studies, except this is even higher.  It is a bit unusual to find such a low number of these could be cultured.  Shigella culture is not sensitive because of lack of enrichment broth, but it is strange to  only recover Shigella from two samples.   
Many thanks for the positive evaluation of our work.

Some specific suggestions:
Line 353-380 The description of the two Shigella isolates is too wordy.  The data is important, but it could be said using many fewer lines.

Done

Regarding the discussion section.  The authors suggest the intestinal bacteria are coming from the oral cavity and are perhaps protected from stomach acid by oral or nasal mucus.   Another explanation might be lack of gastric acid in the stunted children and this may also be associated with Helicobacter infection.  See Gracey M, et al. The stomach in malnutrition. Arch Dis Child 1977;52:325-7 and Windle HJ, et al Childhood Helicobacter pylori infection and growth impairment in developing countries: a vicious cycle? Pediatrics 2007;119:e754-9.  The authors have information on the pH of the stomach (especially if stimulated) or information on rates of infection with Helicobacter in this population?
We did not perform qPCR to assess for *Helicobacter* carriage. Regarding the pH, there are 108 subjects whose SIBO and pH data were available and analyzed in a bivariate analysis. There was no significant association between SIBO and gastric pH:

ttest ph_estomac, by(SIBO)

Two-sample t test with equal variances

------------------------------------------------------------------------------

   Group |     Obs        Mean    Std. Err.   Std. Dev.   [95% Conf. Interval]

---------+--------------------------------------------------------------------

      No |      21    2.142857    .3604608    1.651839    1.390949    2.894765

     Yes |      79    2.646835    .2462167    2.188421    2.156656    3.137015

---------+--------------------------------------------------------------------

combined |     100       2.541    .2089758    2.089758    2.126347    2.955653

---------+--------------------------------------------------------------------

    diff |           -.5039783    .5131575               -1.522323     .514366

------------------------------------------------------------------------------

    diff = mean(No) - mean(Yes)                                   t =  -0.9821

Ho: diff = 0                                     degrees of freedom =       98

    Ha: diff < 0                 Ha: diff != 0                 Ha: diff > 0

 Pr(T < t) = 0.1642         Pr(|T| > |t|) = 0.3285          Pr(T > t) = 0.8358

We now address this in the Discussion section:

“Another explanation might be lack or the reduced concentration of gastric acid in the stunted children in LMICs (i.e. due to *Helicobacter pylori* contamination) which may lead to this bacterial overgrowth since gastric secretions is one of the normal defense mechanisms controlling the intestinal microflora [Ref. 42: Gracey M, et al. The stomach in malnutrition. Arch Dis Child 1977;52:325-7. doi: 10.1136/adc.52.4.325.]. However, the pH values measured in our stunted children were low and there were no significant differences in a bivariate analysis for pH and SIBO (S7 table).”

If the authors are proposing the intestinal bacteria originate from the oral cavity, what are the suggestions for future studies to explore this. Also, how did the intubation method exclude the potential that the fluid obtained was not contaminated by the nasal bacteria as it was passing through the nasal cavity?
Unlikely but we agree and it has been clarified in the MS (as previously requested by reviewer2). The first ml of aspiration from both stomach and duodenum were discarded in order to flash out possible contaminating bacteria and carry-over from the more proximal compartments. Therefore, it seems unlikely that duodenal sampling technique via nasogastric tube could introduce nasopharyngeal flora into the sample thus contaminating it. This question was addressed by both the scientists and the surgeons/MD at the beginning of the study. We added those sentences in the ‘Limitation section’ of the MS (Lines 756-758): Regarding the duodenal sampling technique via nasogastric, we ensured the first ml of aspiration from both stomach and duodenum were discarded in order to flash out possible contaminating bacteria and carry-over from the more proximal compartments. This was also added in the M&M section (Lines 249-251)

The section on limitations is too brief.  Some additional limitations include:  
Done (we added several items, notably on the duodenal sampling technique), as previously explained (responses to reviewer 2)

1) the study did not directly measure evidence of EE, but they assumed this.

Revisited as requested by the two reviewers. We also removed some of the references to EE (or EED).

2) the finding of the bacteria in the small intestine and stool shows an association with malnutrition, but this does not prove cause and effect.  While came first?  
In low- and middle-income countries, the first cause of malnutrition is an inadequate or insufficient diet. Additional factors due to the sanitary conditions the children are living in, such as an over-contamination of the digestive tract by pathobionts or pathogens (bacteria, parasites, viruses) should also play a secondary important role in maldigestion and inflammatory processes (preventing to some extend complementary feeding interventions). Microbial causes of malabsorption include duodenal bacterial overgrowth as addressed in our manuscript but also Whipple's disease (inability to metabolize fats caused by the bacterium *Tropheryma whipplei*), and numerous infections or infestations that are most frequently seen in immunocompromised patients.

Malabsorption syndrome refers to the group of diseases with impairment of digestive and/or absorptive processes arising from structural or functional disorders of the small intestinal tract and its associated organs. For instance, milk and soy protein intolerance, tropical sprue, infective enteritis, autoimmune enteropathy, drug-induced injury and bacterial overgrowth, can cause similar pathological changes in westernized countries. [S R Owens, J K Greenson. 2006 The pathology of malabsorption: current concepts. https://doi.org/10.1111/j.1365-2559.2006.02547.x; Ian Brown. 2012 The Pathology of Malnutrition and Malabsorption. https://doi.org/10.1002/9781118399668.ch21; Crane JR et al. Environmental enteric dysfunction: an overview. https://doi.org/10.1177/15648265150361S113]

3) the study did not attempt to correlate oral and intestinal bacteria.

We agree. That was not done in this study but it was previously done in our first publication [Ref 14, now 15] and in a recent paper submitted to the Journal Gastroenterology [EMID:9fe92c9fb23881d5] (Stunted child growth is associated with small intestinal colonization by oral bacteria driving lipid malabsorption and inflammation by Vonaesch P et al.).

Table 4 should clarify that these are fecal samples

Done. The figure legend of Table 4 now reads as follows:

**“Table 4.** Comparison between stunted children and controls for the presence of bacterial diarrheagenic agents in fecal samples.”

Reviewer #2: OVERALL:  
The authors present an interesting analysis of children in Madagascar investigating the dysbiotic microbiome associated with SIBO using culture methods.  They attempt to determine risk factors for SIBO as well as describe enteric pathogen carriage.  They report 2 Shigella isolates which were sequenced and further investigated.  This analysis has significant overlap with reference 14 and likely utilizes some of the same samples although this is unclear.  Major critiques center around unrelated analyses being reported and unclear methodology.  The introduction and discussion sections are also highly speculative.

We improved the text accordingly. All remarks from the two reviewers were taken into account allowing to substantially improve our manuscript.

We did not really overlap with ref 14 (now 15) which addressed only 153 feces and 12 duodenal aspirates from Madagascan children. This study investigated 464 fecal samples and 109 duodenal aspirates.
The manuscript has been changed as follows:
Line 138: “In a preliminary study (feces from 153 children and 12 duodenal aspirates) [15], we showed …”

Line 142: “The current research aimed to assess SIBO and gut infections in a population of children (N=464) recruited in Antananarivo …”

Line 206: “The case control was extensively described in a preliminary study [15] and is similar to the full-study.”

Further, it seems there is a missed opportunity here to investigate the relationship of enteric pathogen carriage to SIBO.  I would suggest correlating pathogen presence or burden (CT value) with the continuous measure of CFU/ml of growth on duodenal aspirate culture. 

Thank you for the suggestion. We did analyze for a possible association between enteric pathogen carriage and SIBO and added the results into the manuscript (material and methods and results’ sections):

The newly added section read as follows:

**Material and methods**

“Additionally, we correlated the pathogen presence in stools with the CFU/ml values from duodenal aspirates in aerobic and anaerobic culture conditions using the non-parametric Wilcoxon’s rank sum test.”

**Results**

“Using the Wilcoxon’s rank sum test, we correlated the pathogen presence in stools with the CFU/ml values from duodenal aspirates in aerobic and anaerobic culture conditions. The only pathogen significantly associated with the duodenal CFU count was *Campylobacter* spp. (p=0.026) (S4 Table).”

**Table S4**

ABSTRACT:
-The preferred term is Environmental Enteric Dysfunction (EED), not Environmental Enteric Disease.  EE generally stands for Environmental Enteropathy, a term that has gone out of favor in recent years.
We corrected the term “Environmental Enteric Dysfunction” throughout the manuscript.

-While EED has certainly been associated with enteric pathogen carriage, mechanisms of pathogenesis have yet to be fully explained.  Throughout the manuscript, authors should refrain from stating that EED is caused by pathogen carriage.

We agree with the author that there is no established association with any pathogen (yet). The manuscript was reviewed in that sense and now reads as follows:
~~Abstract: Environmental Enteric Dysfunction (EED) which could be caused by a repeated or continuous~~.
Lines 27-31: “Environmental Enteric Dysfunction (EED) refers to an incompletely defined syndrome of inflammation, reduced absorptive capacity, and reduced barrier function in the small intestine. It is widespread among children and adults in low- and middle-income countries and is also associated with poor sanitation, certain gut infections possibly resulting in an abnormal gut microbiota, small intestinal bacterial overgrowth (SIBO) and stunting.”

-EED is a complex syndrome, part of which includes an upper intestinal dysbiosis but to say EED causes a dysbiosis is misleading and unproven. 
We agree.

The manuscript was reviewed in that sense and especially the first passage in lines 27-31 now reads as follows: “Environmental Enteric Dysfunction (EED) refers to an incompletely defined syndrome of inflammation, reduced absorptive capacity, and reduced barrier function in the small intestine. It is widespread among children and adults in low- and middle-income countries and is also associated with poor sanitation, certain gut infections resulting in an abnormal gut microbiota, small intestinal bacterial overgrowth (SIBO) and stunting.”

-Lines 40 – 44 “The presence of those commonly…”:  This sentence is purely speculative without evidence.  Would remove such speculation.
The sentence was deleted.

INTRODUCTION:
-Overall, much of the introduction is author opinion/speculation which is inappropriate in scientific writing.  I suggest the authors focus on reviewing the relevant literature (cited appropriately and correctly) to suggest the current study is needed. 
We have reviewed the Introduction and deleted any speculations/ opinions.

-Line 75:  Adults in LMICs also have evidence of EED although this is less studied.
Thank you for this information but this is not in the scope of our study. Indeed, the earliest descriptions of EED, previously known as ‘Tropical Enteropathy’, date back to the 1960s when an abnormal microscopic appearance of the small bowel was observed in adults from LMICs [Cook GC, Kajubi SK, Lee FD. Jejunal morphology of the African in Uganda. *The Journal of pathology.* Jul 1969;98(3):157-169]. This was also recently addressed in Hossain, M., Begum, S.M.K.N., Rahman, M.M. *et al.* Alterations in the histological features of the intestinal mucosa in malnourished adults of Bangladesh. *Sci Rep* **11,** 2355 (2021). <https://doi.org/10.1038/s41598-021-82079-6>.

-Line 76:  The term “developing world” is no longer preferred.  Recommend using the World Bank classification of low- and middle-income countries.
We agree. We changed it to low and middle-income countries (LMICs).

Lines 86 – 89 “The second, non-exclusive, etiology…”  This entire sentence is unproven speculation.  Citations are inappropriately used and neither discusses oral taxa, taxa associated with SIBO, or SIBO histology.   
The text was fully reworked.

Lines 127-133 now read as follows: “SIBO is defined as the presence of excessive bacteria (greater than 10^5^ CFU/ml) in the small intestine. It is frequently implicated as the cause of chronic diarrhea and malabsorption. SIBO can be measured noninvasively by hydrogen breath testing or by anaerobic and aerobic cultures of endoscopically aspirated upper gastrointestinal fluid [12]. In a study performed on Bangladeshi children, SIBO was associated with a decreased length-for-age Z score since birth and poor sanitation but was independent of frequent or recent diarrheal disease. It was also associated with intestinal inflammation but not with an increased permeability or systemic inflammation [13].”

Authors need to be clear in the introduction as to how this manuscript differs from reference 14 [now 15] and adds something novel to the literature.

We made it clearer in the revised manuscript by mentioning that Ref 15 (previously 14) was performed on a restricted number of samples and did not include any analysis of enteropathogens carriage.
Line 138: “In a preliminary study (feces from 153 children and 12 duodenal aspirates) [15], we showed …”

Line 142: “The current research aimed to assess SIBO and gut infections in a population of children (N=464) recruited in Antananarivo …”

Line 206: “The case control was extensively described in a preliminary study [15] and is similar to the full-study.”

        --------------------

PLOS authors have the option to publish the peer review history of their article (<a href="<https://scanmail.trustwave.com/?c=6967&d=mpCO4WhTB1i45qv1jRgUAoRmlKhSLGjLkZRp0syBxQ&u=https%3a%2f%2fjournals%2eplos%2eorg%2fplosntds%2fs%2feditorial-and-peer-review-process%23loc-peer-review-history>" target="_blank">what does this mean?</a>). If published, this will include your full peer review and any attached files.<br><br>

If you choose “no”, your identity will remain anonymous but your review may still be made public.<br><br>

<b>Do you want your identity to be public for this peer review?</b> For information about this choice, including consent withdrawal, please see our <a href="<https://scanmail.trustwave.com/?c=6967&d=mpCO4WhTB1i45qv1jRgUAoRmlKhSLGjLkc1m1cSEwA&u=https%3a%2f%2fwww%2eplos%2eorg%2fprivacy-policy>" target="_blank">Privacy Policy</a>.

Reviewer #1: No

Reviewer #2: No


Figure Files:
While revising your submission, please upload your figure files to the Preflight Analysis and Conversion Engine (PACE) digital diagnostic tool, <https://scanmail.trustwave.com/?c=6967&d=mpCO4WhTB1i45qv1jRgUAoRmlKhSLGjLkc1p0pbYyQ&u=https%3a%2f%2fpacev2%2eapexcovantage%2ecom> PACE helps ensure that figures meet PLOS requirements. To use PACE, you must first register as a user. Then, login and navigate to the UPLOAD tab, where you will find detailed instructions on how to use the tool. If you encounter any issues or have any questions when using PACE, please email us at [figures@plos.org](mailto:figures@plos.org).

Data Requirements:
Please note that, as a condition of publication, PLOS' data policy requires that you make available all data used to draw the conclusions outlined in your manuscript. Data must be deposited in an appropriate repository, included within the body of the manuscript, or uploaded as supporting information. This includes all numerical values that were used to generate graphs, histograms etc.. For an example see here: <http://scanmail.trustwave.com/?c=6967&d=mpCO4WhTB1i45qv1jRgUAoRmlKhSLGjLkZE428PVxw&u=http%3a%2f%2fwww%2eplosbiology%2eorg%2farticle%2finfo%3adoi%2f10%2e1371%2fjournal%2epbio%2e1001908%23s5>

Reproducibility:
To enhance the reproducibility of your results, we recommend that you deposit your laboratory protocols in <http://scanmail.trustwave.com/?c=6967&d=mpCO4WhTB1i45qv1jRgUAoRmlKhSLGjLkZQ4hsbZlA&u=http%3a%2f%2fprotocols%2eio> where a protocol can be assigned its own identifier (DOI) such that it can be cited independently in the future. Additionally, PLOS ONE offers an option to publish peer-reviewed clinical study protocols. Read more information on sharing protocols at <https://scanmail.trustwave.com/?c=6967&d=mpCO4WhTB1i45qv1jRgUAoRmlKhSLGjLkZBs1MyGlw&u=https%3a%2f%2fplos%2eorg%2fprotocols%3futm%5fmedium%3deditorial-email%26utm%5fsource%3dauthorletters%26utm%5fcampaign%3dprotocols>

__________________________________________________
In compliance with data protection regulations, you may request that we remove your personal registration details at any time.  (Use the following URL: <https://scanmail.trustwave.com/?c=6967&d=mpCO4WhTB1i45qv1jRgUAoRmlKhSLGjLkcA_gMTWkA&u=https%3a%2f%2fwww%2eeditorialmanager%2ecom%2fpntd%2flogin%2easp%3fa%3dr%29> Please contact the publication office if you have any questions.
